# Baculovirus Actin Rearrangement-Inducing Factor 1 Can Remodel the Mammalian Actin Cytoskeleton

**DOI:** 10.1128/spectrum.05189-22

**Published:** 2023-02-13

**Authors:** Anika Steffen, Björn Reusch, Nadine Gruteser, Daniela Mainz, Renza Roncarati, Arnd Baumann, Theresia E. B. Stradal, Dagmar Knebel-Mörsdorf

**Affiliations:** a Department of Cell Biology, Helmholtz Centre for Infection Research, Braunschweig, Germany; b Center for Biochemistry, University Hospital Cologne, University of Cologne, Cologne, Germany; c Institute of Biological Information Processing, Molecular and Cellular Physiology, Research Center Juelich, Juelich, Germany; d Department of Pediatrics, University Hospital Cologne, University of Cologne, Cologne, Germany; Center for Research and Advanced Studies

**Keywords:** Arif-1, AcMNPV, F-actin remodeling, actin dynamics, B16-F1 cells, TN368 cells

## Abstract

The actin rearrangement-inducing factor 1 (Arif-1) of Autographa californica multiple nucleopolyhedrovirus (AcMNPV) is an early viral protein that manipulates the actin cytoskeleton of host insect cells. Arif-1 is conserved among alphabaculoviruses and is responsible for the accumulation of F-actin at the plasma membrane during the early phase of infection. However, the molecular mechanism underlying Arif-1-induced cortical actin accumulation is still open. Recent studies have demonstrated the formation of invadosome-like structures induced by Arif-1, suggesting a function in systemic virus spread. Here, we addressed whether Arif-1 is able to manipulate the actin cytoskeleton of mammalian cells comparably to insect cells. Strikingly, transient overexpression of Arif-1 in B16-F1 mouse melanoma cells revealed pronounced F-actin remodeling. Actin assembly was increased, and intense membrane ruffling occurred at the expense of substrate-associated lamellipodia. Deletion mutagenesis studies of Arif-1 confirmed that the C-terminal cytoplasmic region was not sufficient to induce F-actin remodeling, supporting that the transmembrane region for Arif-1 function is also required in mammalian cells. The similarities between Arif-1-induced actin remodeling in insect and mammalian cells indicate that Arif-1 function relies on conserved cellular interaction partners and signal transduction pathways, thus providing an experimental tool to elucidate the underlying mechanism.

**IMPORTANCE** Virus-induced changes of the host cell cytoskeleton play a pivotal role in the pathogenesis of viral infections. The baculovirus Autographa californica multiple nucleopolyhedrovirus (AcMNPV) is known for intervening with the regulation of the host actin cytoskeleton in a wide manner throughout the infection cycle. The actin rearrangement-inducing factor 1 (Arif-1) is a viral protein that causes actin rearrangement during the early phase of AcMNPV infection. Here, we performed overexpression studies of Arif-1 in mammalian cells to establish an experimental tool that allows elucidation of the mechanism underlying the Arif-1-induced remodeling of actin dynamics in a well-characterized and genetically accessible system.

## INTRODUCTION

Many viruses encode gene products that redirect the structure and function of the host actin cytoskeleton to support penetration, intracellular movement during entry and egress, viral transcription and replication, and cell-to-cell spread (1). An open question is how virus-mediated remodeling of the actin cytoskeleton could also alter cellular functions in host organisms, which in turn may contribute to infection at the organismal level. The arthropod-specific virus Autographa californica multiple nucleopolyhedrovirus (AcMNPV) of the genus *Alphabaculovirus* (family *Baculoviridae*) is an enveloped double-stranded DNA virus which infects most tissues of lepidopteran larvae and induces an extensive change of actin dynamics in cultured insect cells. During infection, F-actin assembly occurs in a cascade-like fashion, which starts with the formation of early cytoplasmic actin cables and finally results in the assembly of nuclear F-actin, which is required for nucleocapsid morphogenesis (2–7). Intriguingly, actin-based motility not only contributes to the passage of nucleocapsids through the cytoplasm upon entry but also is involved in the transit of newly assembled nucleocapsids to the nuclear periphery (8, 9).

A further step of actin remodeling early in infection is the accumulation of F-actin clusters at the plasma membrane induced by the actin rearrangement-inducing factor 1 (Arif-1) (2, 10). While Arif-1 is not a component of budded viruses (11, 12), the gene is strongly expressed from 4 to 12 h postinfection (p.i.), and its promoter activation depends on the viral transcription factor IE1 (10, 11). Arif-1 resides at the place of action within the plasma membrane and contains three predicted transmembrane domains (11). So far, homologous sequences of *arif-1* are only known for baculoviruses. While it is absent in granuloviruses, the *arif-1* gene (AcMNPV orf20) is highly conserved among nucleopolyhedroviruses, which may be relevant to the differences in occlusion body morphology, tissue tropism, and cytopathology among the two genera of baculoviruses (13). Intriguingly, Arif-1 is much more highly expressed in Trichoplusia ni midgut tissue than in a cultured *T. ni* cell line, suggesting that it may be important in establishing secondary infection in the hemocoel (14). Indeed, studies in larvae infected with genetically modified Bombyx mori nucleopolyhedrovirus (BmNPV) lacking Arif-1 indicated that Arif-1 slows down systemic infection (15). Moreover, recent findings in cultured Spodoptera frugiperda cells showed that Arif-1 induces the formation of invadosome-like structures which can accelerate infection in larvae by penetrating the basal lamina and thereby assisting passage of the virus from the midgut to the tracheal system (16). In the mammalian system, invadosomes are known as actin-based structures that are involved in the proteolytic invasion of cells (17). In cell culture, Arif-1 has a redundant function, since in both permissive *S. frugiperda* and TN-368 cells, infection with Arif-1-mutated AcMNPV does not harm virus transport, genome replication, or progeny production (11).

To further understand how Arif-1 contributes to the efficiency of infection in larval tissue, it is essential to unravel the mechanism underlying its function of manipulating the actin cytoskeleton. Here, we overexpressed Arif-1 in mouse melanoma B16-F1 cells, a frequently used model system to study cell motility, lamellipodia, and adhesion formation (18, 19). Cell migration and substrate adhesion require dynamic reorganization of the actin cytoskeleton, which leads to cellular projections, such as lamellipodia, filopodia, and membrane ruffles, which in turn rely on *de novo* nucleation and polymerization of actin into filaments (20). Our results demonstrated that Arif-1 can induce a pronounced remodeling of the actin cytoskeleton in B16-F1 cells, including exaggerated formation of membrane ruffles at the expense of lamellipodia. Overexpression of Arif-1 in TN368 cells confirmed a comparable change of actin dynamics in murine and insect cells. Our observations demonstrated that the mode of Arif-1 action relies on conserved interactions with actin-binding proteins or with components of actin regulatory signaling pathways.

## RESULTS AND DISCUSSION

### Arif-1-induced remodeling of the actin cytoskeleton in TN368 cells.

**FIG 1:**
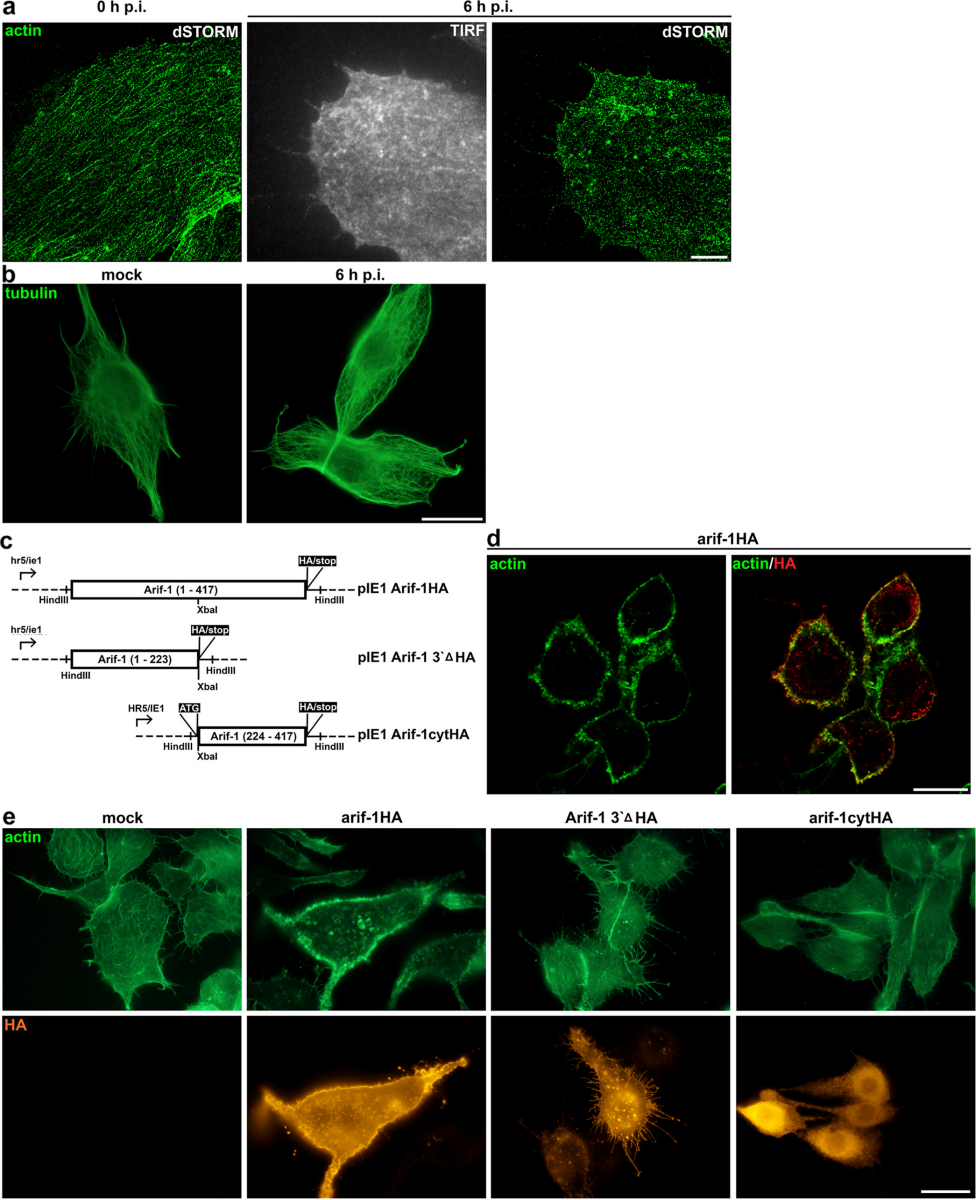
Actin rearrangement in TN368 cells after AcMNPV infection or Arif-1 expression. (a) After seeding TN368 cells on poly-d-lysine-coated coverslips, cells were infected with AcMNPV at a multiplicity of infection of 10 PFU/cell. Representative superresolution images (*n* = 3 independent experimental settings) are shown after adding the virus suspension (0 h p.i.) and 6 h p.i. Phalloidin staining was used to visualize the actin filament network at 0 h p.i. and the actin accumulations at 6 h p.i. Scale bar, 5 μm. (b) After mock infection and at 6 h p.i., α-tubulin immunostainings indicated a comparable microtubule network. Infected cells were detected by IE2 staining (not shown). (c) Schematic illustrating the Arif-1HA-tagged constructs expressing full-length *arif-1* ORF or truncated versions under the control of the *hr5/ie1* promoter. (d) TN368 cells seeded on poly-d-lysine-coated coverslips were transfected with the construct pIE1Arif-1HA (arif-1HA) for 48 h (*n* > 3). The overlay of a confocal bottom section with HA (red) and phalloidin (green) staining depicts the accumulation of actin clusters, which colocalized with Arif-1 at the plasma membrane. (e) After transfection of Arif-1 constructs, cells expressing the N-terminal Arif-1 (arif-1 3′Δ HA) or C-terminal Arif-1 (arif-1cytHA) showed an actin cytoskeleton (green) comparable to that of mock-transfected cells, while cells expressing full-length Arif-1 (arif-1HA) demonstrated the loss of actin stress fibers and accumulation of F-actin clusters. HA stainings (orange) allowed visualization of localization of full-length and N-terminal Arif-1 (arif-1 3′ΔHA) at the plasma membrane, while the C-terminal Arif-1 (arif-1cytHA) localized in the cytoplasm (*n* = 3). Scale bars, 20 μm.

Previous infection studies with Arif-1 mutant AcMNPV had demonstrated that Arif-1 expression is responsible for F-actin accumulation, mainly at the plasma membrane, at 3 to 7 h p.i. (11). Here, we reexamined the Arif-1-induced effects during infection and upon transient overexpression in the highly permissive insect cell line TN368 in more detail. Prior to infection, superresolution microscopy (dSTORM) revealed a rather dense network of fine actin filaments with a calculated fiber width of 40 to 50 nm (Fig. 1a). The distinct actin rearrangement at 6 h p.i. included the replacement of filaments by F-actin dots of various sizes, which were found not only in the cytoplasm but also near and at the plasma membrane, as shown by total internal reflection fluorescence (TIRF) imaging (Fig. 1a). Moreover, the formation of membrane protrusions including filopodia was observed (Fig. 1a). Based on Arif-1 mutant viruses, we previously demonstrated the causal link of these steps of actin remodeling and Arif-1 expression (10, 11). To exclude an additional effect of Arif-1 on the tubulin network at this stage, we stained infected TN368 cells for β-tubulin, and this indicated no change at 6 h p.i. compared to mock-infected cells (Fig. 1b). Progressive reorganization of microtubules later in infection is known to correspond with infection-induced cell rounding (21).

To investigate actin remodeling upon transient expression of Arif-1, we fused the 3′ end of the *arif-1* open reading frame (ORF) with a hemagglutinin (HA) tag into a vector allowing expression under the control of the early viral *hr5/ie1* promoter to optimize expression and detection of Arif-1, compared to previously used constructs under the control of the pe38 promoter (10) (Fig. 1c). Upon expression in TN368 cells for 48 h, Arif-1 localized mainly at the plasma membrane, where colocalization with actin clusters was visualized by confocal imaging (Fig. 1d). Furthermore, Arif-1 expression induced actin accumulations in the proximity of the nucleus and strongly affected the network of stress fibers (Fig. 1e).

Previous structural predictions and localization studies have suggested the presence of up to three transmembrane domains in the N terminus of Arif-1 and a C-terminal region extending into the cytoplasm (11). A functional role of the C-terminal 200 amino acids for the Arif-1-induced actin changes was initially shown by infection studies with mutant AcMNPV lacking this domain (11). Recent expression studies of C-terminal truncations of Arif-1 have indicated that the transmembrane domains are also required for Arif-1 function and that the C terminus between residues 303 and 417 is at least necessary for the formation of invadosome clusters in *S. frugiperda* cells (16). Here, we expressed either the N- or C-terminal domain of Arif-1 in TN368 cells (Fig. 1c). Our observations revealed that the N terminus of Arif-1 (pIE1Arif-1 3′ΔHA) localized at the plasma membrane and intracellular clusters, while the Arif-1 C terminus (pIE1Arif-1cytHA) showed a cytoplasmic localization (Fig. 1e). Expression of neither the N nor the C terminus induced the changes in F-actin structures characteristic for full-length Arif-1, supporting that the C terminus has to be linked to transmembrane regions located in the N terminus to exert its effects (Fig. 1e). However, changes such as enhanced filopodia formation induced by the N-terminal Arif-1 could occasionally be observed (Fig. 1e). Our results were consistent with the finding that invadosome formation also requires the membrane-targeted C terminus of Arif-1 (16).

Taken together, transient expression of Arif-1 in TN368 cells mimicked changes of actin dynamics that are visible during infection as a second step of actin arrangement. The characteristic alterations of the actin cytoskeleton encompass F-actin accumulations at the plasma membrane and the loss of stress fibers, while perinuclear actin clusters were mainly observed in Arif-1-expressing cells. As recently reported, we also observed a lack of Arif-1-induced invadosome-like structures in TN368 cells (16).

### Transient expression of Arif-1 in B16-F1 cells strongly changed actin dynamics.

**FIG 2:**
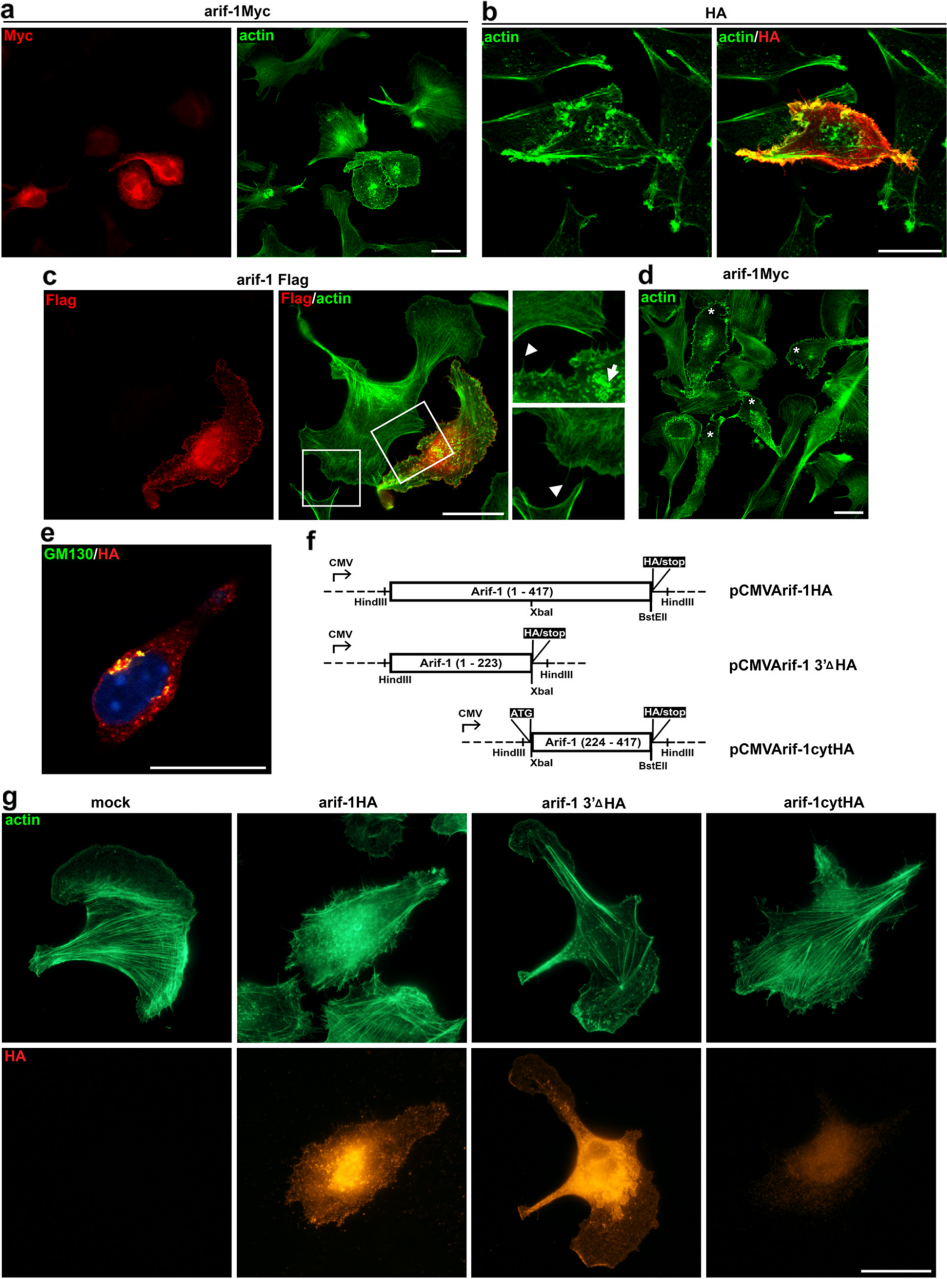
Arif-1-induced actin rearrangement in B16-F1 cells. B16-F1 cells transfected with Myc-, HA-, or Flag-tagged Arif-1 constructs for 24 h were seeded on laminin-coated coverslips. (a) Phalloidin staining (green) showed membrane ruffles at the entire cell periphery and the loss of lamellipodia and stress fibers in an Arif-1-expressing cell visualized by Myc immunostaining (red) (*n* > 3). (b) The confocal bottom section depicts colocalization of HA-tagged Arif-1 (red) and F-actin (green) at the plasma membrane (*n* = 2). (c) Flag immunostaining (red) allowed visualization of Arif-1 in the perinuclear space and at the plasma membrane, while phalloidin staining (green) depicted a rather jagged cell periphery with multiple filopodia (magnified image, arrowheads) compared to the untransfected cell and actin clusters in the cytoplasm (magnified image, arrow) (*n* > 3). (d) Myc-tagged Arif-1-expressing cells (asterisks) showed altered actin (green) cytoskeleton architecture, including reduced stress fibers, cytoplasmic actin clusters, and membrane ruffles (*n* > 3). (e) Confocal section from the middle, showing partial colocalization of *cis*-Golgi structures visualized by GM130 staining (green) and perinuclear HA-tagged Arif-1 (red) (*n* = 3). (f) Schematic illustrating the Arif-1HA-tagged constructs expressing the full-length *arif-1* ORF or truncated versions under the control of the CMV promoter. (g) Strong actin (green) accumulations were visible after full-length Arif-1 expression, while the N and C termini of Arif-1 showed an actin network comparable to that in mock-transfected cells. A mock-transfected cell with characteristically actin-dense lamellipodium is shown. HA stainings (orange) allowed visualization of localization of full-length and N-terminal Arif-1 (arif-1 3′ΔHA) at the plasma membrane, while the C-terminal Arif-1 (arif-1cytHA) localized in the cytoplasm (*n* = 3). Scale bars, 20 μm.

Baculoviruses cannot infect mammalian cells to produce viral progeny. However, the entry process and the delivery of the AcMNPV genome via the nuclear pore of AcMNPV might be identical to the process in insect cells, suggesting that some virus-host cell interactions are conserved (22). To explore whether Arif-1 exhibited a conserved mode of action in mammalian cells, we expressed Arif-1 in murine B16-F1 melanoma cells, a very-well-characterized cell system for actin-based motility. B16-F1 cells efficiently spread and migrated on laminin, whereby in polarized migration, these cells mostly employ wide lamellipodia formed parallel to the substratum (19). We performed transient expression studies with a vector containing the *arif-1* open reading frame extended with an HA, Flag, or Myc tag under the control of the cytomegalovirus (CMV) promoter. Initial observations revealed that Arif-1 indeed induced actin remodeling in various mammalian cell lines from different species, including epithelial canine kidney cells (MDCK), the fibroblast-like African green monkey kidney cell line COS-1, mouse embryonic fibroblasts, the human fibroblast cell line Wi26, and the murine melanoma cell line B16-F1. The characteristic common feature of the Arif-1-induced actin rearrangement in all cell lines was the loss of stress fibers and ectopic formation of actin accumulations, whereas the extent, size, and distribution of these F-actin clusters showed some variations. To further investigate changes of actin dynamics, we chose B16-F1 cells as a model for detailed analyses. Upon transient expression of Arif-1 for 24 h, cell morphology in Arif-1-expressing cells was altered independently of the type of tag; flat lamellipodia were frequently lost and replaced by small membrane ruffles, which were not routinely observed in untransfected B16-F1 cells (Fig. 2a, b, c, and d). Membrane ruffles were found along the entire cell periphery, leading to a loss of cell polarity (Fig. 2a). Cytoplasmic actin filament bundles were diminished and replaced by F-actin clusters, which accumulated not only at the plasma membrane but also in proximity of the nucleus (Fig. 2a and c). Arif-1-expressing cells displayed various extents of actin rearrangements, with reduced presence of actin bundles and altered morphology compared to untransfected cells, often showing loss of cell polarity and, most frequently, perinuclear actin clusters and membrane ruffles instead of lamellipodia (Fig. 2d, asterisk). In cells expressing small amounts of Arif-1, only perinuclear staining was detected, suggesting that translocation to the plasma membrane was rather inefficient at low expression levels (data not shown). Costaining of Arif-1 and the Golgi component GM130 demonstrated partial colocalization of perinuclear Arif-1 with *cis*-Golgi structures (Fig. 2e). Furthermore, Arif-1 colocalized with F-actin to various degrees at the plasma membrane and also with cytoplasmic F-actin clusters (Fig. 2b and c). As previously demonstrated for AcMNPV-infected TN368 cells (11), Western blot analyses supported membrane association of Arif-1 in transfected B16-F1 cells (data not shown).

To address the role of N- and C-terminal parts of Arif-1 in B16-F1 cells, we generated N- or C-terminally truncated versions of Arif-1 under the control of the CMV promoter (Fig. 2f). Upon transfection of both truncated Arif-1 variants, stress fiber formation was maintained and no alterations of cell morphology, rearrangement of F-actin, or additional actin accumulations were observed (Fig. 2g). The staining pattern of the truncated Arif-1 proteins was comparable to those in transfected TN368 cells; expression of the N terminus (pCMVArif-1 3′ΔHA) showed intracellular staining and localization at the plasma membrane, while the C terminus (pCMVArif-1cyt HA) was localized in the cytoplasm (Fig. 2g). When full-length Arif-1 was expressed, a rather strong Arif-1 staining correlated with a complete loss of fibers and strong actin accumulations throughout the cytoplasm (Fig. 2g).

**FIG 3:**
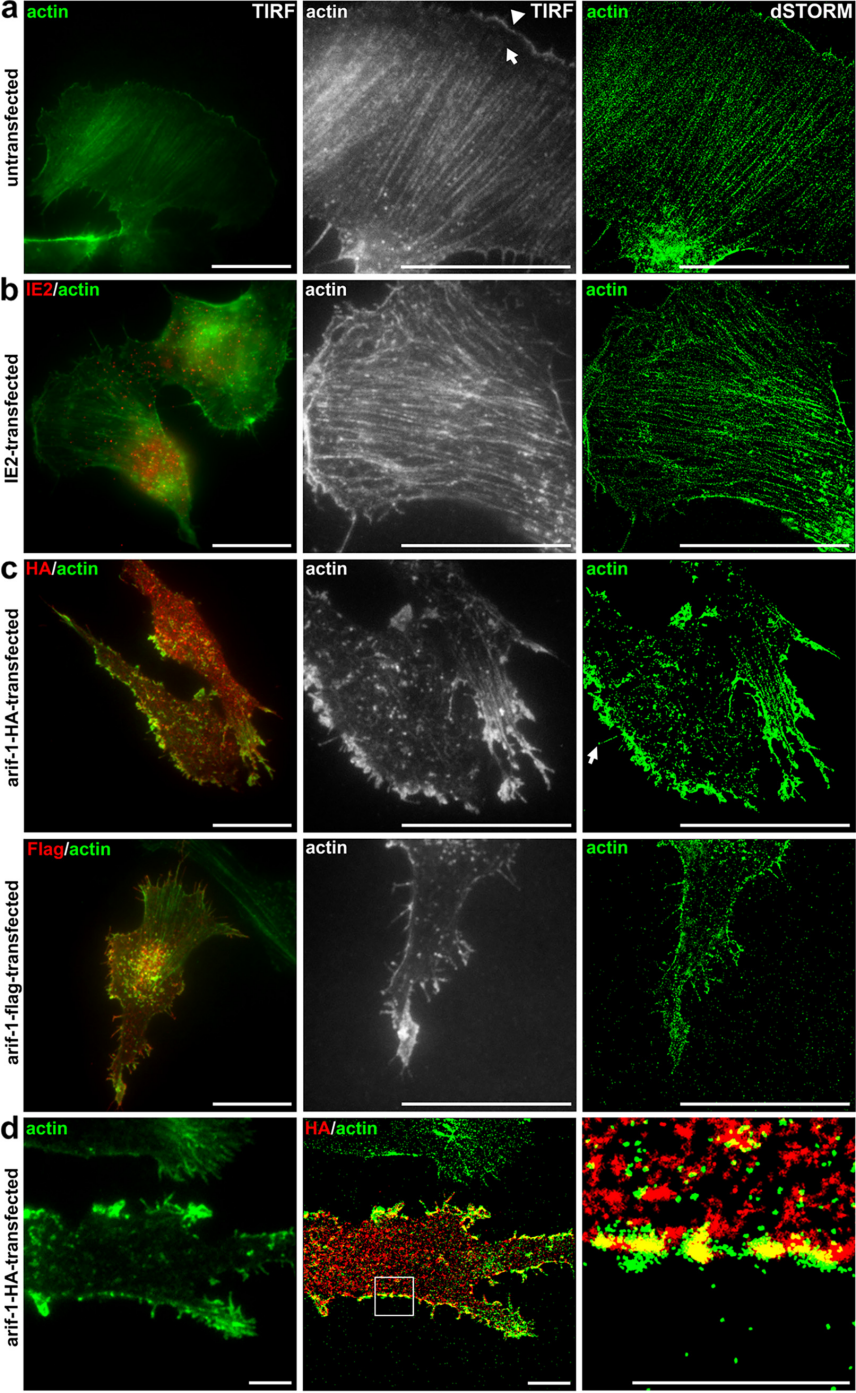
Superresolution microscopy of Arif-1-induced actin rearrangement in B16-F1 cells. B16-F1 cells transfected with IE2-, HA-, or Flag-tagged Arif-1 constructs for 24 h were seeded on laminin-coated coverslips. HA or Flag immunostainings allowed visualization of Arif-1, F-actin was stained with phalloidin, and IE2 was detected with anti-IE2 antiserum (*n* = 3). (a) The untransfected cell showed the fiber network with lamellipodium (arrowhead) and lamella (arrow) at the front and stress fibers at the rear. (b) The stress fiber network was maintained in the IE2-expressing cell. (c) Filopodia (arrow) and strong actin clusters were visible at the plasma membrane, while smaller clusters and dots were detected in the cytoplasm. (d) The magnified merged dSTORM image shows discontinuous colocalization of Arif-1 aggregates and actin clusters at the plasma membrane and partial colocalization of Arif-1 and actin dots in the cytoplasm. Scale bars, 20 μm (a, b, and c) or 5 μm (d).

For comparison with the remodeled F-actin structures in TN368 cells, Arif-1-induced changes in B16-F1 cells were more closely analyzed by TIRF microscopy and reconstructed superresolution images (dSTORM). Untransfected cells showed the characteristic actin filament network in a polarized cell with thicker stress fibers at the rear and thinner filaments in the lamella (Fig. 3a). To visualize stress-induced unspecific responses to the transfection procedure, we transiently expressed IE2, an immediate-early gene of AcMNPV with nuclear localization (23, 24). Although some minor changes of the lamellipodia were rarely observed, stress fibers were maintained (Fig. 3b). Arif-1-expressing cells, however, lost their polarity, and lamellipodia and stress fibers nearly disappeared (Fig. 3c). The various sizes of F-actin clusters showed colocalization with Arif-1 predominantly at the plasma membrane but also at small intracellular clusters (Fig. 3c and d). Next to membrane ruffles, filopodia could be observed (Fig. 3c, arrow). The Arif-1-induced actin rearrangements represented features that are untypical for B16-F1 cells, which normally show wide and smooth lamellipodia (Fig. 2c and g). Intriguingly, the loss of stress fibers and the accumulation of F-actin clusters mainly at the plasma membrane were reminiscent of infected TN368 cells at 6 h p.i. (Fig. 1a).

**FIG 4:**
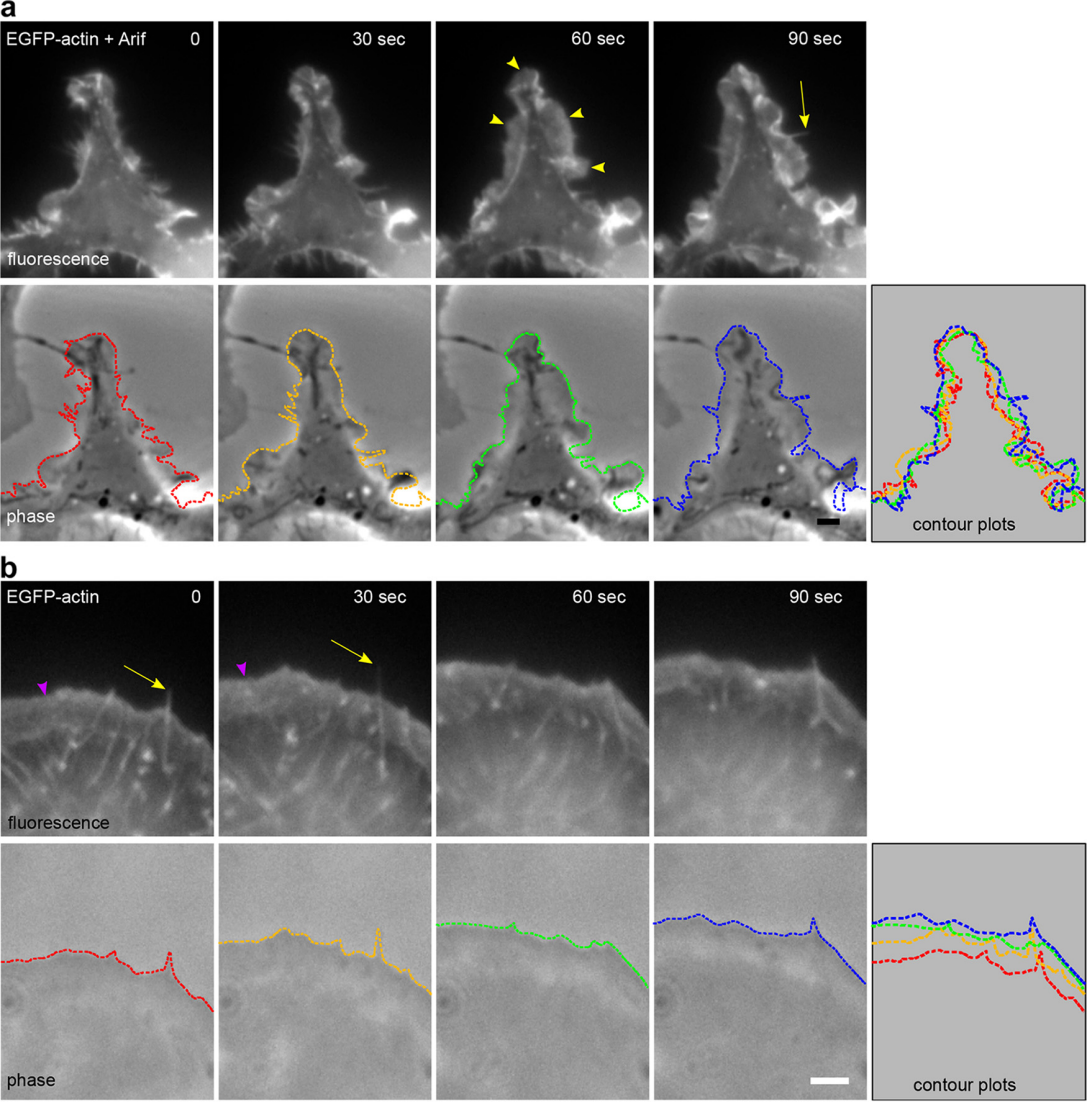
Arif-1-induced actin dynamics in B16-F1 cells. Time-lapse images of a B16-F1 cell seeded on laminin-coated coverslips and coexpressing EGFP-actin and Myc-tagged Arif-1 or expressing EGFP-actin alone. The upper panel shows wide-field fluorescence images of the time-lapse movie at the indicated time points, and the lower panel shows the corresponding phase-contrast images. The cell periphery is outlined with colored dashed lines and overlaid in the contour plot. (a) An Arif-1-expressing cell showed dynamic ruffles (yellow arrowheads) and filopodia (yellow arrow). The very dynamic ruffle formation did not lead to a net translocation, as shown by the contour plot. (b) Characteristics of the EGFP-actin-transfected cell included a broad, effectively protruding lamellipodial network (magenta arrowhead) harboring embedded bundles termed microspikes, some of which occasionally developed into filopodia (yellow arrows). The contour plot illustrates the continuous protrusion of the cell edge. Scale bars, 2 μm.

To visualize how the Arif-1-induced remodeling of the actin cytoskeleton can change the dynamics of cell motility, we performed time-lapse microscopy with B16-F1 cells cotransfected with EGFG-actin and Arif-1 constructs. Strikingly, Arif-1 expression led to highly dynamic membrane protrusions, including ruffles and filopodia, suggesting an active impact on F-actin polymerization and depolymerization processes (Fig. 4a). In contrast to the mock-transfected cell, where the lamellipodium was continuously protruding (Fig. 4b), the dynamic ruffle formation in the Arif-1-expressing cell could not be translated into active net protrusion, as illustrated by a contour plot (Fig. 4a). Taken together, Arif-1-induced changes of the actin cytoskeleton in B16-F1 cells were reminiscent of actin changes observed in Arif-1-expressing or AcMNPV-infected TN368 cells, supporting that Arif-1 targets highly conserved components of the actin regulatory machinery.

### Summary and conclusion.

Transient expression studies in B16-F1 and TN368 cells demonstrated that Arif-1 expression could convert stress fibers into distinct actin accumulations in both insect and mammalian cells. This conserved function was only executed in the presence of both N and C termini. Arif-1-induced changes in B16-F1 cells led to disturbed cell polarity and strongly modified actin dynamics, which in turn influenced actin-based motile processes, such as cell edge protrusion. Because B16-F1 cells represent a well-established model to unravel the pathways and interaction partners underlying cell migration and adhesion, they offered a unique tool to unravel the molecular mechanisms of Arif-1-induced actin remodeling and analysis of the interaction partners. This is a precondition to understand its role on the organismal level during infection of the larval host.

## MATERIALS AND METHODS

### Cells, viruses, and infection.

Trichoplusia ni TN368 cells (25) were grown in TC100 medium (26) supplemented with 10% fetal calf serum (FCS) at 27°C. Prior to infection or transfection, TN368 cells were seeded on coverslips coated with poly-d-lysine (100 μg/mL) to enhance adhesion. As TN368 cells can vary in the strength of substrate adhesion, we tested two different cell sources, which demonstrated only minor differences in the extent of Arif-1-induced F-actin changes. The murine melanoma cell line B16-F1 (ATCC CRL-6323) was maintained in Dulbecco modified Eagle medium (4.5g/liter glucose; Life Technologies) supplemented with 10% FCS and 2 mM l-glutamine (Thermo Fisher Scientific) at 37°C and imaged in sterile-filtered imaging medium (nutrient mixture F-12/HAM, 10% FBS, 1 mM l-glutamine, 100 U/mL penicillin-streptomycin).

Infection of TN368 cells was performed with AcMNPV plaque isolate E (27) as wild-type virus. The time point when the virus inoculum was added to the cells was labeled 0 h p.i. To exclude stress-induced changes of the actin cytoskeleton upon addition of the virus inoculum, cells were fixed shortly after addition of viruses as a control (Fig. 1a). After 1 h of incubation at 27°C, the virus inoculum was replaced with medium.

### Plasmid constructions.

Plasmid CMVArif-1myc was generated by insertion of a PCR-amplified fragment, including the *arif-1* ORF in the vector pcDNA3.1(+) version C (Life Technologies). The PCR fragment was amplified from plasmid pArif-1 (10) using the forward primer 5′-GGGGGAAGCTTGCCACC ATG TTA AAT AAA ATC ACT GCA-3′ and the reverse primer 5′-GGGGGGTGACCATC ATC ATA AAC GGG TAA-3′, with a HindIII site at the 5′ end and a BstEII site at the 3′ end. After cleavage of pCMVArif-1myc with BstEII, the oligonucleotides, including a Flag tag (5′-GGTCACC AC GAT TAC AAG GAT GAC GAC GAT AAG TAG AAGCTT GGTCACC-3′) or an HA tag-encoding sequence (5′-GGTCACC AC TAT CCA TAT GAT GTT CCA GAT TAT GCA TAG AAGCTT GGTCACC-3′), each followed by a stop codon, were inserted and resulted in pCMVArif-1Flag and pCMVArif-1HA, respectively (Fig. 2e). To excise the C terminus of Arif-1, pCMVArif-1HA was cleaved with XbaI and BstEII, followed by insertion of the oligonucleotide 5′-CT AGA TAT CCA TAT GAT GTT CCA GAT TAT GCA TAG AAGCTT G-3′. The resulting pCMVArif-1HA-3′Δ included only the N-terminal 223 codons of Arif-1 (Fig. 2f). For expression studies in insect cells, the 3′ deletion mutant of HA-tagged Arif-1 was cloned under the control of the *hr5/ie1* promoter. Plasmid pCMVArif-1HA3′Δ was cleaved with HindIII, and the HindIII fragment was inserted in pIE1/hr5/PA (gift from Paul Friesen, University of Wisconsin—Madison), which included the *hr5* enhancer/*ie1* promoter and p35 poly(A) signal in a Bluescript backbone. The resulting plasmid was labeled pIE1Arif-1HA3′Δ (Fig. 1c). To generate pIE1Arif-1HA (Fig. 1c), pCMVArif-1HA was cleaved with HindIII, and the HindIII fragment was inserted in pIE1/hr5/PA. The PCR fragment including the N-terminal deletion mutant of Arif-1 was amplified from pIE1Arif-1HA using the forward primer 5′-GGGGGAAGCTTCAAA ATG TTG TAC GTG CAG TTA AAG GAG ATG CG-3′ and the reverse primer 5′-TGTTGAGTGCACTAGTCGAGGT-3′. The amplified fragment was inserted in pIE1/hr5/PA, resulting in pIE1Arif-1HAcyt (Fig. 1c). In addition, the amplified fragment was inserted in pCMVArif-1HA after the *arif-1* ORF was excised by cleavage with HindIII, resulting in pCMVArif-1HAcyt (Fig. 2f). The plasmids pCMVArif-1HAcyt and pIE1Arif-1HAcyt contained only the C-terminal 193 codons of Arif-1 (Fig. 1c and 2f). Plasmids pEGFP-actin (Clontech) and pCMV-IE2 (24) have been described previously.

### Transfection experiments.

TN368 cells were seeded on poly-d-lysine-precoated coverslips in TC100 supplemented with 10% FCS and grown overnight at 27°C. DNA was diluted in Opti-MEM (Gibco), and X-tremeGENE HP DNA transfection reagent (Merck) was added, followed by incubation for 20 min at room temperature according to the manufacturer’s instructions. After adding the transfection mix, cells were incubated for 48 h at 27°C.

B16-F1 cells were transfected in a 24-well plate or 3-cm dish using JetPrime transfection reagent (PolyPlus) and incubated for 10 min at room temperature according to the manufacturer’s instructions. After overnight incubation at 37°C, transfected cells were seeded on coverslips precoated with laminin (Sigma catalog number L2020) at 25 μg/mL in laminin coating buffer (150 mM NaCl, 50 mM Tris-HCl; pH 7.4) and incubated for 4 h at 37°C (28).

### Immunocytochemistry.

Infected or transfected TN368 cells were fixed with 4% formaldehyde for 20 min, permeabilized with 0.1% Triton X-100 for 10 min, and stained with rat anti-HA (monoclonal antibody clone 3F10; 1:1,000; Roche) at room temperature. For staining of microtubules, cells were fixed with methanol (Sigma) for 5 min at –20°C, permeabilized with 0.1% Triton X-100 for 15 min, and stained with mouse anti-α-tubulin (1:250; Calbiochem) and rabbit anti-IE2 antiserum (1:2,000) (23) at room temperature. Transfected B16-F1 cells were fixed with 2% or 4% formaldehyde for 20 min, permeabilized with 0.1% Triton X-100, and stained with mouse anti-myc (monoclonal antibody clone 9E10; 1:100; Sigma-Aldrich), rat anti-HA (monoclonal antibody clone 3F10; 1:1,000; Roche), mouse anti-Flag (monoclonal antibody clone M2; 1:250; Sigma-Aldrich), and mouse anti-GM130 (monoclonal antibody clone 35; 1:50; BD Biosciences) for 60 min at room temperature, followed by incubation with species-specific Alexa Fluor-conjugated secondary antibodies and (4′,6-diamidino-2-phenylindole) for 45 or 60 min at room temperature. F-actin staining was performed with Alexa Fluor 488-conjugated phalloidin (Invitrogen). Microscopy was performed using a wide-field epifluorescence microscope (Zeiss Axiophot) equipped with a Nikon digital sight camera system (DS-2MV) and NIS Elements software, a Leica DM IRB/E microscope linked to a Leica TCS-SP/5 confocal unit, or a Zeiss Axiovert system equipped with a Photometrics CoolSnap HQ2 camera (Photometrics). Images were assembled using Photoshop (Elements 2018; Adobe), Metamorph 7.10 (Molecular Devices), and Inkscape 0.92.

In addition, infected TN368 cells and transfected B16-F1 cells for analysis by superresolution microscopy were fixed with 4% formaldehyde, permeabilized with 0.5% Triton X-100 for 10 min, blocked with 5% normal goat serum for 30 or 45 min, and incubated with primary antibodies for 60 min, followed by incubation with the corresponding secondary antibodies for 60 min at room temperature. After washing, cells were again fixed with 4% formaldehyde for 5 min. F-actin was stained with phalloidin ATTO 488 (Sigma) for 60 min at room temperature, followed by additional fixation with 4% formaldehyde for 5 min to preserve the staining.

### Superresolution microscopy.

Superresolution microscopy was performed in TIRF mode using a home-built setup with an Olympus IX-71 inverted microscope body, an iXonEM+ DU897E (Andor) camera, and 405-, 488-/514-, 561-, and 642-nm lasers. An achromatic lens, used to focus the expanded laser beams on the back aperture of the objective (oil immersion objective, 60×, ApoN TIRF, numerical aperture 1.49; Olympus), was mounted on a movable stage to change focus position on the objective back aperture to switch between wide-field and TIRF mode (29–31). dSTORM (30, 32) was performed in imaging buffer (90 mM β-mercaptoethylamine, 5% [wt/vol] glucose, 5% [vol/vol] glycerol, in phosphate-buffered saline; pH 7.4) and 20 μL oxygen-scavenging solution [0.1% (wt/vol) glucose oxidase, 0.12% (vol/vol) catalase, 50% (vol/vol) glycerol, 4 mM Tris(2-carboxyethyl)phosphine, 52 mM KCl, 20 mM Tris-HCl; pH 7.5]. A stack of 4,000 to 12,000 images was recorded. The program RapidStorm (33) was used for image reconstruction with high sensitivity for circular spots and an appropriate threshold for background suppression.

### Time-lapse microscopy.

Transfected B16-F1 cells were replated onto laminin-coated 15-mm coverslips, incubated for at least 4 h, and then mounted in a temperature-heated Warner chamber (PH-4; Warner Instruments) in imaging medium at 37°C. Cells were captured every 10 to 15 s by wide-field fluorescence and phase-contrast optics using a Zeiss Axiovert system equipped with fluorescein isothiocyanate filters and a Photometrics CoolSnap HQ2 camera driven by VisiView software (Visitron Systems) (28).
